# Laparoscopic uterosacral ligament suspension: a systematic review and meta-analysis of safety and durability

**DOI:** 10.3389/fsurg.2023.1180060

**Published:** 2023-06-07

**Authors:** Carlo Ronsini, Francesca Pasanisi, Stefano Cianci, Maria Giovanna Vastarella, Marika Pennacchio, Marco Torella, Alfredo Ercoli, Nicola Colacurci

**Affiliations:** ^1^ Department of Woman, Child and General and Specialized Surgery, University of Campania “Luigi Vanvitelli,” Naples, Italy; ^2^ Department of Gynaecology and Obstetrics, University of Campania “Luigi Vanvitelli,” Naples, Italy; ^3^ Obstetrics and Gynecology Unit, Department of Human Pathology of Adult and Childhood “G. Baresi,” University Hospital “G. Martino”, Messina, Italy

**Keywords:** uterosacral ligament suspension, laparoscopy, minimally invasive surgery, mini-invasive surgery (MIS), vaginal, safety

## Abstract

**Introduction:**

Pelvic organ prolapse (POP) is a widespread condition affecting from 40% to 60% of women. Reconstructive vaginal surgeries are the most commonly performed procedures to treat POP. Among those, uterosacral ligament suspension (USLS), which is usually performed transvaginally, preserves pelvic statics and dynamics and appears to be an effective method. Laparoscopic USLS is a valid alternative to vaginal approach, and the aim of our review is to confirm its safety and feasibility and to compare clinical outcomes among the procedures.

**Materials and methods:**

Following the recommendations in the Preferred Reporting Items for Systematic Reviews and Meta-Analyses (PRISMA) statement, we systematically searched the PubMed and Scopus databases in December 2022. We made no restriction on the publication year nor on the country. Data about POP-Q recurrence rate (RR), intraoperative and postoperative complications (graded according to Clavien–Dindo classification), readmission rate, and reoperation rate were collected and analyzed. We used comparative studies for meta-analysis.

**Results:**

A total of nine studies fulfilled inclusion criteria: two articles were non-comparative retrospective observational studies, three more articles were comparative studies where laparoscopic USLS was confronted with other surgical techniques (only data of laparoscopic USLS were analyzed), and four were comparative retrospective cohort studies between laparoscopic and vaginal USLS procedures. The comparative studies were enrolled in meta-analysis. Patients were analyzed concerning perioperative risks and the risk of recurrence. The meta-analysis highlighted that there was no clear inferiority of one technique over the other.

**Discussion:**

Laparoscopic USLS is a technique with a low complication rate and low recurrence rate. Indeed, laparoscopic procedure allows better identification of anatomical landmarks and access to retroperitoneum. Moreover, efficacy over time and durability of Laparoscopic (LPS) USLS was also observed. However, these data should be weighed in light of the length of follow-up, which was in a very short range. Further, focused and prospective studies will be necessary to confirm this finding.

## 1. Introduction

Pelvic organ prolapse (POP) is a highly prevalent phenomenon that is expected to affect approximately 40%–60% of women during their lifetime. These numbers are predicted to increase throughout the coming years (1, 2). The most commonly undertaken procedures for the treatment of POP are reconstructive vaginal interventions. The main techniques involve plicating the damaged connective tissue or fascia with sutures and resuspending the uterus or vagina to firm up the supporting structures, such as the uterosacral or sacrospinous ligaments, or the pubic bone or sacrum. Surgery can be performed transvaginally or abdominally (open or minimally invasive procedures) (3). According to the DeLancey theory, endopelvic fascia is the base of the statics and dynamics of pelvic visceral support. Proper interaction and integrity of structures of Levels I and II is diriment in order to provide support and physiological function of the pelvic organs (4). On this line, uterosacral ligament suspension (USLS) allows both statics and support on the one side and preserves organ functions and physiological interactions on the other. Thus, evidences suggest that it represents an effective surgical procedure (5). The vaginal approach for uterosacral ligament suspension (V-USLS) is the most common procedure to restore apical support preserving the orientation of the vaginal axis in its natural position (6). Laparoscopic approach to uterosacral ligament suspension (L-USLS) is now frequently adopted to improve visualization and decrease the rate of injury to contiguous structures such as the ureters, vessels, rectum, and sacral nerves. It is suitable for the treatment of younger women with uterine descent that, eventually, allows the preservation of the uterus (7, 8). The aim of this study is to analyze perioperative outcomes, in terms of complications and risk of recurrence and reoperation, of laparoscopic USLS procedure. In addition, we intended to differentiate the results of comparative studies for vaginal vs. laparoscopic procedure, in order to establish safety and feasibility of the latter.

## 2. Materials and methods

The methods for this review and meta-analysis were specified *a priori* based on the recommendations in the Preferred Reporting Items for Systematic Reviews and Meta-Analyses (PRISMA) statement (9). We registered the review to the PROSPERO site for meta-analysis with protocol number CRD42023400398.

### 2.1. Search method

We performed a systematic search for articles concerning the safety and feasibility of laparoscopic USLS alone and comparing laparoscopic and vaginal USLS approach in the treatment of POP. The PubMed and Scopus databases were screened in December 2022, and no restriction on the publication year nor on the country was considered. Only entirely English published studies were enrolled. Search imputes were “Laparoscopy [MeSH Term] OR laparoscopic surgery [Word Text] AND Shull [Word Text] OR uterosacral ligament suspension [Word Text] OR culdoplasty [Word Text].”

### 2.2. Study selection

**Figure 1:**
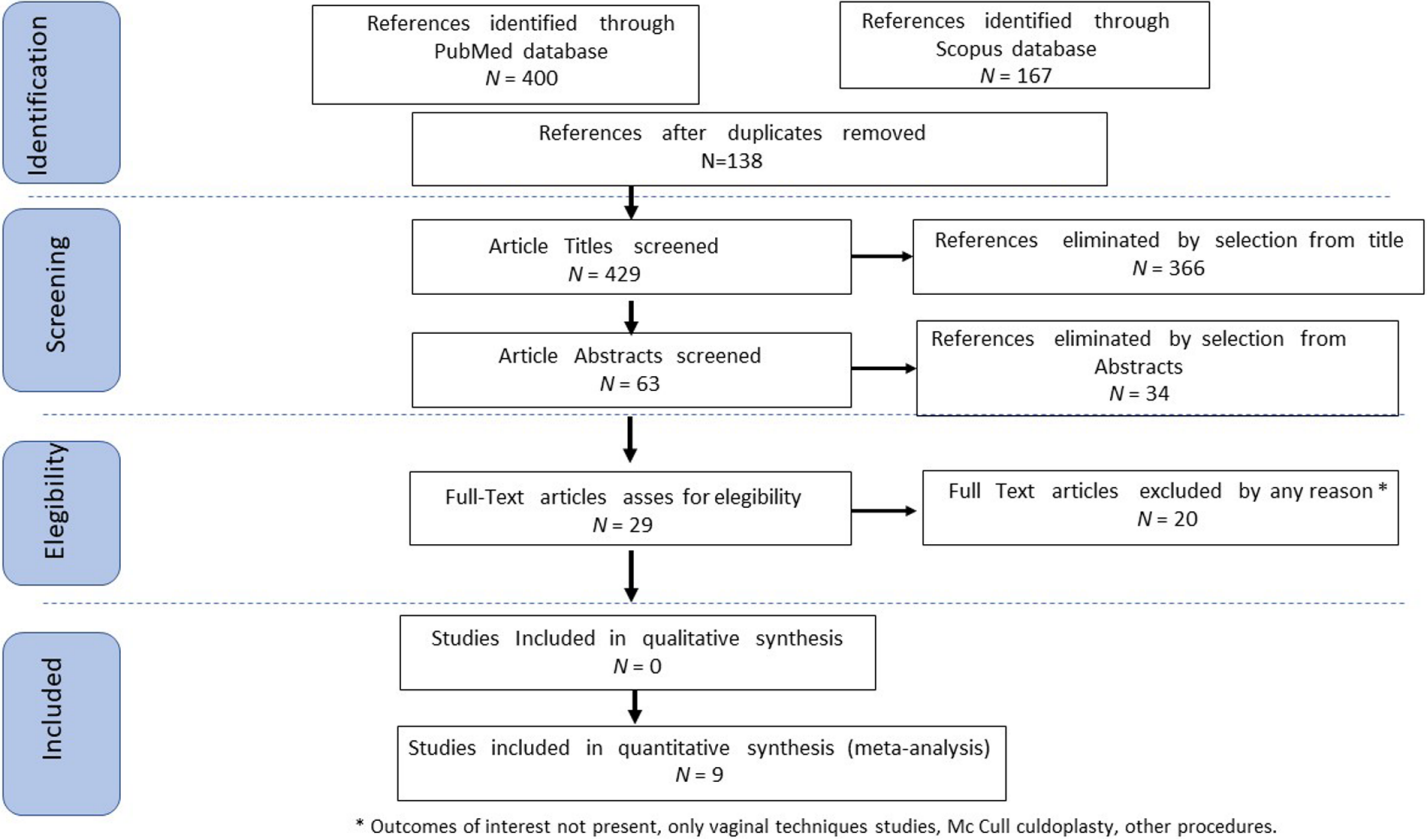
PRISMA flowchart.

Study selection was independently conducted by FP and MP. In case of discrepancy, CR decided on inclusion or exclusion criteria. The inclusion criteria were as follows: (1) studies that included patients with symptomatic utero-vaginal prolapse treated with USLS via laparoscopic route; (2) studies that compared laparoscopic and vaginal USLS techniques for the treatment of utero-vaginal prolapse; (3) studies that reported at least one outcome of interest: POP-Q recurrence rate (RR), intraoperative and postoperative complications (graded according to Clavien–Dindo classification), readmission rate, and reoperation rate (RoR); and (4) originally published peer-reviewed articles. Non-original studies, preclinical trials, animal trials, abstract-only publications, and articles in a language other than English were excluded. If possible, the authors of studies that were only published as congress abstracts were contacted via email and asked to provide their data. The studies selected and all reasons for exclusion are mentioned in the PRISMA flowchart (Figure 1). All included studies were assessed regarding the potential conflicts of interest.

### 2.3. Data extraction

FP and MP extracted the data from all relevant series and studies. Data regarding laparoscopic USLS surgical times and outcomes, mean follow-up (FUP), and intra- and postoperative complications were collected and analyzed and eventually liken to vaginal USLS data in comparative studies in order to assess the safety and feasibility of laparoscopic route alone and its comparison to vaginal technique.

### 2.4. Statistical analysis

Heterogeneity among the studies was tested using the Chi-square test and I-square test (10). The risk rate (RR) and 95% confidence interval (CI) were used for dichotomous variables. Fixed-effects models conducted statistical analysis without significant heterogeneity (*I*^2 ^< 50%) or random-effect models if *I*^2 ^> 50%. Disease Free Survival (DFS) and Overall Survival (OS) were used as clinical outcomes. In each study, recurrence was defined as the presence of a POP-Q prolapse of ≥II after surgery. Reoperation was defined as repeated urogynecological surgery for any reason. Readmission was defined as rehospitalization in the early first 30 days after surgery for any reason. A complication was considered as any event according to Clavien–Dindo classification (11). Chi-square tests were used to compare continuous variables. Subgroup analysis was performed in patients with Clavien–Dindo complication grade of ≤2 or ≥3. Review Manager version 5.4.1 (RevMan 5.4.1) and IBM Statistical Package for Social Science version 25.0 (IBM SPSS 25.0) for MAC were used for statistic calculation. For all performed analyses, a *p*-value of <0.05 was considered significant.

### 2.5. Quality assessment

We assessed the quality of the included studies using the Newcastle–Ottawa scale (NOS) (12). This assessment scale uses three broad factors (selection, comparability, and exposure), with the scores ranging from 0 (lowest quality) to 8 (best quality). Two authors (MP and MT) independently rated the study's quality. Any disagreement was subsequently resolved by discussion or consultation with NC. We used a funnel plot analysis to assess publication bias. We used Egger's regression test to determine the asymmetry of funnel plots.

## 3. Results

### 3.1. Studies’ characteristics

Following the database search, 429 articles on laparoscopic USLS, once duplicates removed, were screened and selected. Afterward, records with no full text, lack of outcomes of interest, and wrong study designs (e.g., reviews or case report) were excluded. At the end of this selection, nine studies were suitable for eligibility and matched the inclusion criteria: two articles were single-armed, non-comparative retrospective observational studies, which evaluate the surgical procedure and outcomes of laparoscopic uterosacral ligament suspension (13, 14); four studies were comparative retrospective cohort studies that analyze the data about laparoscopic and vaginal USLS (15–18); and three more articles were comparative studies where laparoscopic USLS was confronted with other surgical techniques (19–21). From the aforementioned articles, we only extracted data about patients that had undergone laparoscopic USLS and laparoendoscopic single-site ULSL (Figure 1). The countries where the studies were conducted, the publication year range, the design of the studies, the FUP, and the number of participants are summarized in Table 1. The quality of all studies was assessed by the NOS (12). Overall, the publication years ranged from 2014 to 2021. In total, 587 patients who underwent laparoscopic USLS were enrolled. For laparoscopic procedures, the follow-up ranged from 1 to 24 months and for vaginal surgeries from 3 to 26 months. POP-Q stage before surgery was >≠ II on average for all groups.

**Table 1:** Studies’ characteristics.

Author, year	Country	Study design	Year range	No. of participants	Compared technique	Median FUP (months)	Stage of POP-Q
Filmar 2014 (19)	USA	Retrospective cohort study, monocenter	2010–2011	29	261 laparoscopic ASC	3.8	II
Barbier 2015 (15)	USA	Retrospective cohort study, multicenter	2008–2013	148	60 vaginal USLS	3.6	≥I
Turner 2015 (16)	USA	Retrospective cohort study, monocenter	2011–2014	54	119 vaginal USLS	5.3	≥II
Davila 2016 (20)	UK	Retrospective cohort study, multicenter	2011–2016	13	5 RSS USLS	18	II–III
Houlihan 2018 (17)	Canada	Retrospective cohort study, monocenter	2014–2016	54	152 vaginal USLS	21.1	≥I
Chill 2020 (21)	Israel	Retrospective cohort study, monocenter	2010–2019	70	49 vaginal mesh culposuspension	3.8	≥II
Ma 2020 (13)	China	Retrospective observational study, monocenter	2016–2019	57	–	3	≥II
Sezgin 2021 (18)	Turkey	Case–control study, monocenter	2015–2020	37	37 vaginal USLS	12	≥II
Panico 2021	Italy	Retrospective observational study, monocenter	2026–2018	60	–	24	>II

FUP, follow-up; ASC, abdominal sacral colpopexy; LESS, laparoendoscopic single site; RSS, robotic single site; USLS, uterosacral ligament suspension.

### 3.2. Outcomes

The outcomes of the main studies are presented in Tables 2–4. The data concerning laparoscopic USLS were extracted and analyzed from the comparative studies. Only studies presenting at least one outcome of interest were included. Table 2 describes the laparoscopic USLS operative outcomes, related to the surgical technique. Filmar 2014 (19) did not report any outcome of interest. The mean operative time, including port placement, is 120.6 min (range 60–190.1 min). The mean blood loss is 106.8 mL (range 50–200 mL). Chill 2020 (21) and Sezgin 2021 (18) were the two studies that had not analyzed the former data. Overall, intraoperative complications are described only in two cases in Turner 2015 (16) with a rate of 2.7%, including six cases of ureteral injury recognized intraoperatively and treated by stent placement. The mean hospitalization is 1.8 days (range 1–3).

**Table 2:** Operative outcome laparoscopic USLS.

Author, year	Median operative time (min)	Median estimated blood loss (mL)	Intraoperative complications (%)	Mean hospitalization (days)
Barbier 2015 (25)	NR	137.5	0	1
Turner 2015 (16)	190.1	101.3	2.7	1
Davila 2016 (20)	144	82	NR	2
Houlihan 2018 (17)	127.9	200	0	2
Chill 2020 (21)	87.3	NR	0	2
Me 2020 (13)	60	50	NR	3
Sezgin 2021 (18)	115	NR	0	2
Panico 2021	120	70	NR	2

NR, not reported; mL, milliliters; min, minutes.

**Table 3:** Safety outcome laparoscopic USLS.

Author, year	Postoperative complication G1−G2^a^ (%)	Type	Postoperative complication G3−G5^a^ (%)	Type
Filmar 2014 (19)	27.5	3 dyspareunia 2 granulation tissue 2 de novo SUI 1 cystotomy	0	–
Barbier 2015 (15)	9.5	13 UTI within 6 weeks 1 transfusion	NR	–
Turner 2015 (16)	78.3	33 urinary retention 8 UTI	24.1	13 major complications
Davila 2016	30.7	2 SUI 1 fever 1 urinary tract infection	8.0	1 umbilical hernia
Houlihan 2018 (17)	51.8	13 infection 12 dyspareunia 2 painful intercourse 1 transfusion	NR	–
Chill 2020 (21)	21.5	1 SUI 11 urge incontinence	0	–
Ma 2020 (13)	1.8	1 fever	0	
Sezgin 2021 (18)	0	–	0	–
Panico 2021	26.6	16 moderate pain and discomfort	0	

^a^ According to Clavien–Dindo classification.

NR, not reported; SUI, stress urinary incontinence; UTI, urinary tract infection.

**Table 4:** Recurrence of POP outcome laparoscopic USLS.

Author, year	Recurrence of POP (%)	Reoperation (%)
Barbier 2015 (15)	3.9	2.7
Turner 2015 (16)	16.2	1.9
Davila 2016	8	NR
Houlihan 2018 (17)	23.8	2.7
Chill 2020 (21)	5.8	2.9
Panico 2021	16.7	NR

NR, not reported; POP, pelvic organ prolapse.

Postoperative complications are described in Table 3 as safety outcomes. They are divided in G1−G2 (low grade) and G3−G5 (medium/high grade) according to Clavien−Dindo classification (11). A total of 124 cases of low-grade postoperative complications have been counted over 587 patients (21.1%) in all studies. Among those, the most frequently reported are urogenital symptoms. Namely, 33 cases of urinary retention are described in Turner 2015 (16), 12 cases of dyspareunia and two painful intercourse in Houlihan 2018 (17), 11 cases of urge incontinence and one de novo stress urinary incontinence (SUI) are reported in Chill 2020 (21), three cases of dyspareunia and two de novo SUI in Filmar 2014 (19), and two SUI in Davila 2016 (20). Postoperative infections were also common complications, in particular 13 cases including wound, skin, or urinary tract infections in Houlihan 2018 (17), and urinary tract infections (UTI), in particular 13 cases in Barbier 2015 (15), eight in Turner 2015 (16), and one in Davila 2016 (20). Only two studies reported high-grade complications. Davila 2016 (20) reported one case of umbilical hernia, and Turner 2015 (16) reported 13 cases of major complications, among those only one patient suffered from ureteral injury discovered postoperatively and required readmission and reoperation. Overall, grade 3–4 postoperative complication rate is 2.4%.

In six studies we also evaluated data about the recurrence of POP, which is defined as prolapse at or beyond the hymen after primary surgery (Table 4). The mean RR was 12.4%. In four of them (21, 16, 17, 15), extracting data concerning RoR was also feasible, and, on average, 2.5% of patients underwent a second surgery, for either surgical complications or retreatment of POP.

### 3.3. Direct comparison with other techniques

Four studies directly compared laparoscopic USLS and vaginal USLS. In Barbier 2015 (15), at baseline, no statistically significant differences in median FU (3.6 vs. 3.3 months, 0.331) and stage of POP-Q before surgery were reported. Ureteral compromises occurred in six cases in the vaginal group (0.0% vs. 10.0%, respectively, Laparoscopic (LPS) vs. vaginal; *p* < 0.001). A lower median blood loss in the laparoscopic group (137.5 vs. 200.0 mL, respectively; *p* = 0.002) and a lower rate of readmission (0.7% vs. 6.7%, respectively; *p* = 0.025) were identified. Moreover, no other significant differences in postoperative complications between the two groups were found. In Turner 2015 (16), 54 L-USLS and 119 V-USLS procedures with a median follow-up of 5.3 months in both groups were noted. After correcting for concomitant procedures, the operative times of the two approaches were not significantly different (adjusted OR: 1.00, 95% CI: 0.99–1.00). Moreover, no significant difference in complications between groups were reported (24.1% vs. 21.8%, *p* = 0.75). Houlihan 2018 (17) compared 152 patients who had undergone vaginal USLS (V-USLS) and 54 laparoscopic USLS (L-USLS). No statistically significant differences in the mean case time, postoperative length of stay, or perioperative infection were found. Only in the V-USLS group that 14 cases of ureteral obstructions occurred (0% in LPS USLS vs. 9% in V-USLS, *p* = 0.023). Postoperative urinary retention was higher for V-USLS (31% vs. 15%, *p* = 0.024). Recurrence rate of symptomatic prolapse was higher in the V-USLS group (41% vs. 24%, *p* = 0.046); however, retreatment rate was similar in the two groups (0% vs. 7%, *p* = 0.113). In Sezgin 2021 (18), L-USLS and V-USLS groups were similar in distribution, age, BMI, and comorbidities with no significant statistical difference. The mean follow-up was 12 months for both groups. Moreover, no intraoperative complications occurred, while, postoperatively, only one case of ureteral injury was found in a patient in the vaginal group that required a double-J catheter insertion (*p* = 0.327). Operation time, length of hospital stay, and POP-Q stages did not differ in the two groups (*p* > 0.05). No data concerning recurrence of prolapse and readmission were reported.

### 3.4. Meta-analysis

**Figure 2:**
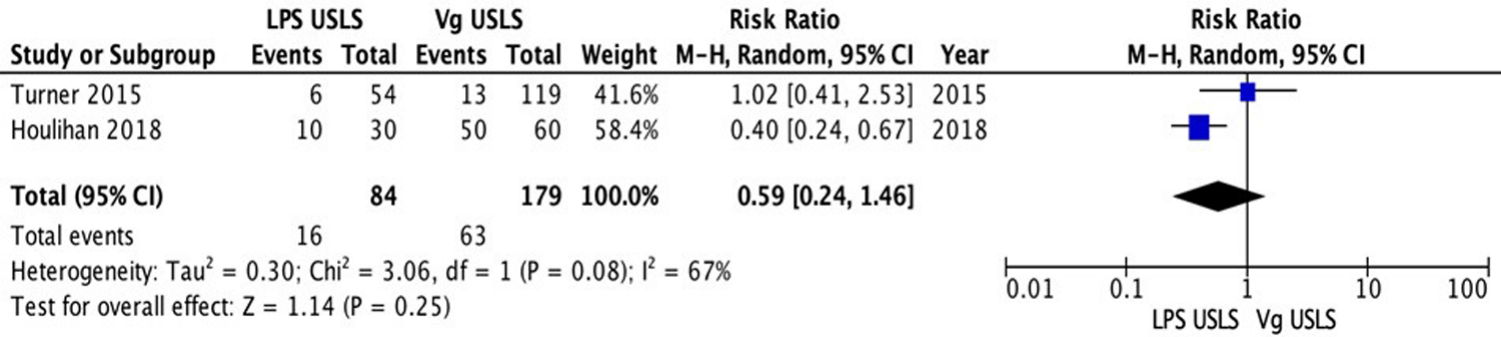
Recurrence rate.

**Figure 3:**
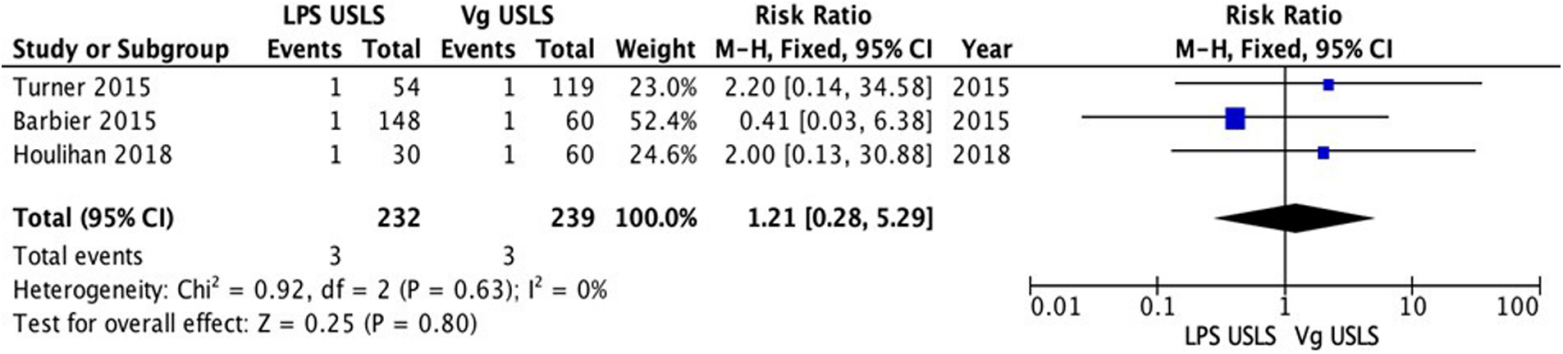
Reoperation rate.

**Figure 4:**
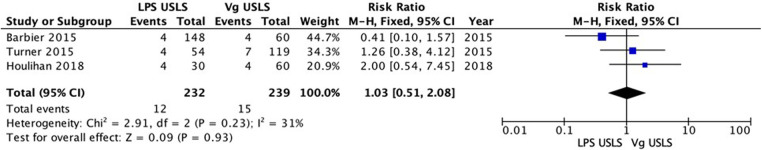
Readmission rate.

**Figure 5:**
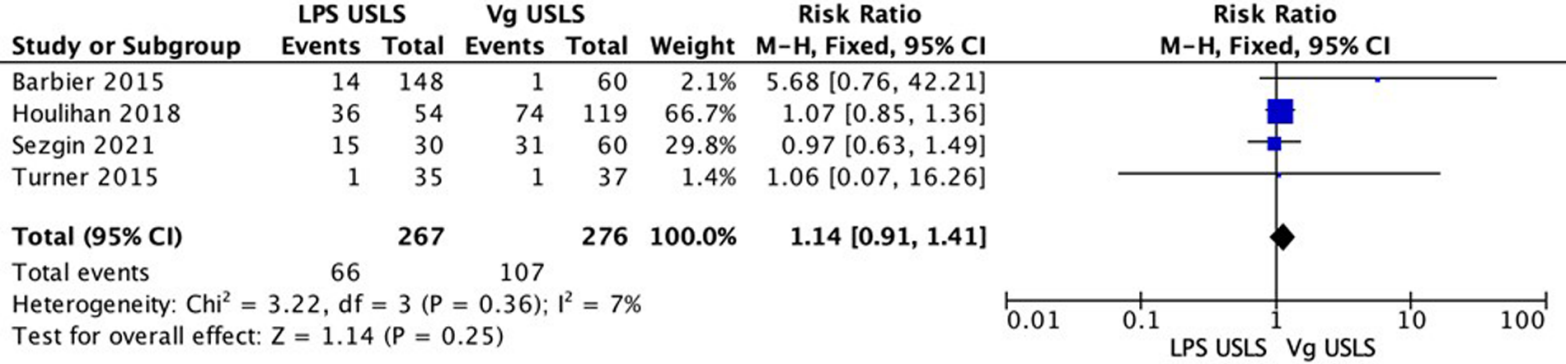
Complication of ≤2*. *According to Clavien–Dindo classification.

**Figure 6:**
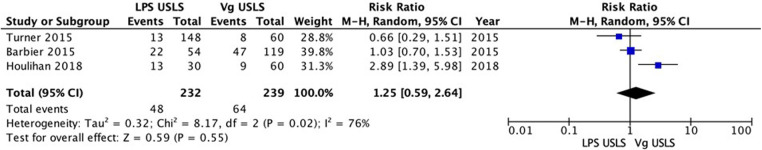
Complication of ≥3*. *According to Clavien–Dindo classification.

The comparative studies comparing LPS USLS and vaginal USLS were enrolled in the meta-analysis. A total of 543 patients were analyzed. Two hundred sixty-seven patients in the LPS USLS arm were compared with the 276 patients who underwent vaginal USLS. Two studies reported data about recurrences. A total of 263 patients were analyzed: 84 in the LPS USLS arm and 179 in the vaginal USLS arm. Sixteen recurrences occurred in the LPS USLS group, while 63 recurrences in the vaginal USLS group. Because of the high heterogeneity (*I*^2^ = 67%; *p* = 0.08), a random-effects model was used. The LPS USLS group showed a non-significant lower risk for recurrences than the vaginal USLS group [RR: 0.59 (95% CI: 0.24–1.46) *p* = 0.25] (Figure 2). We performed a second analysis for the reoperation rate. Three comparative studies were reporting valuable data. Two hundred and thirty-two patients for the LPS USLS group and 239 for the Vaginal USLS group. Because of the low heterogeneity (*I*^2 ^= 31%; *p* = 0.08), a fixed-effects model was used. In this analysis, LPS USLS showed a non-significant similar risk for reoperation compared with vaginal USLS [RR: 1.03 (95% CI: 0.51–2.08) *p* = 0.93] (Figure 3). Another analysis of the readmission rate was conducted on the same group of patients. Because of the low heterogeneity (*I*^2 ^= 0%; *p* = 0.63), a fixed-effects model was used. In this analysis, LPS USLS showed a non-significant higher risk for readmission than vaginal USLS [RR: 1.21 (95% CI: 0.28–5.29) *p* = 0.80] (Figure 4). All patients were finally analyzed for the risk of minor complications (Clavien–Dindo classification of ≤2) and major complications (Clavien–Dindo classification of ≥3). Sixty-six minor complications occurred in the LPS USLS group, while 107 in the vaginal USLS group. Because of the low heterogeneity (*I*^2 ^= 7%; *p* = 0.36), a fixed-effects model was used. The LPS USLS group showed a non-significant higher risk for minor complications than the vaginal USLS group [RR: 1.14 (95% CI: 0.91–1.41) *p* = 0.25] (Figure 5). Further, 48 major complications occurred in the LPS USLS group, while 64 in the vaginal USLS group. Because of the high heterogeneity (*I*^2 ^= 76%; *p* = 0.02), a random-effects model was used. The LPS USLS group showed a non-significant higher risk for major complications than the vaginal USLS group [RR: 1.25 (95% CI: 0.59–2.64) *p* = 0.55) (Figure 6).

## 4. Discussion

LPS USLS is a widely used method for genital prolapse correction (22). With regard to its description, it has been compared with the vaginal approach, considering the gold standard. Already previously, several authors have addressed the performance of prolapse correction. Diwan et al. (23) compared 25 patients who underwent vaginal USLS after hysterectomy with 25 patients who underwent LPS USLS after uterine suspension. They found similar improvement in posterior and anterior prolapse between the groups, with significantly superior apical correction and vaginal length among the LPS USLS group. Other studies have shown similar effects of the LPS USLS compared with the vaginal approach. Lin et al. (24) conducted a retrospective case series of 133 patients who underwent an LPS USLS, with a success rate of 87.2%, similar to the traditional vaginal approach. Nevertheless, the data are much more heterogeneous regarding their safety and durability profiles. Therefore, our review focused on the perioperative outcomes and the risk of recurrence. As highlighted, the technique involves an intraoperative risk close to 0 and remarkably rapid hospitalization times (range 1–3 days). The rate of postoperative complications was also found to be particularly low. Quantitative analysis, although not statistically significant, also showed that LPS USLS does not represent an increased risk for minor complications compared with the vaginal route [grade ≤2 according to Clavien–Dindo classification (11)]. Regarding major complications [grade ≥3 according to Clavien–Dindo classification (11)], LPS USLS appears to only minimally increase the risk and in a non-statistically significant manner [RR: 1.25 (95% CI: 0.59–2.64) *p* = 0.55]. On the other hand, it also appears to show a decreasing trend in the rate of recurrence of genital prolapse grade 2 or higher [RR: 0.59 (95% CI: 0.24–1.46) *p* = 0.25]. In our opinion, this could be attributable to easier identification of anatomic landmarks during the laparoscopic approach. This would allow the uterosacral ligament to be isolated up to its origin, allowing a more significant excursion of the correction. This increases the amount of tissue offered as an anchor and a lengthening of the vaginal length (25). L-USLS does not necessarily need the use of mesh placement in the treatment of prolapse. This is an additional argument in favor of laparoscopy, since a mesh-less treatment of utero-vaginal prolapse avoids complications related to the use of mesh (26). In addition, laparoscopy also allows access to the retroperitoneum. This step is often an integral part of the surgical technique of hysterectomy and offers the advantage of isolating and visualizing the course of the ureter (27). Ureteral damage represents the most common complication during USLS (28). Barber et al. (29) reported an incidence of ureteral damage of 11% in vaginal USLS. Only ureteral damage was reported in the studies reviewed in our review, confirming this hypothesis. Laparoscopic USLS appears to be safer than vaginal USLS in terms of ureteral injury incidence; moreover, in case of ureteral obstruction secondary to vaginal suspension, laparoscopy is an effective option to manage the complication (30). Further speculation must then be made as to whether or not the uterus should be preserved. Hysterectomy may or may not be performed during LPS USLS. The clinical practice guidelines underscore that preservation of the uterus, provided that the surgical approach is the same, helps save time and reduce blood loss in many cases. However, when hysterectomy is considered, the vaginal route is still considered the least morbid, generally resulting in less blood loss and shortest operative time (31–34). Our review also adds insight into the efficacy over time and durability of LPS USLS. The reported recurrence rates ranged from 5.8% to 23.8%, consistent with those reported in the literature for the vaginal technique (35). However, these data should be weighed in light of the length of follow-up, which was in a very short range (3–24 months). In our opinion, the rate of recurrence and reintervention should be the most reliable data on the efficacy of the technique. However, articles often dwell on the success rate, which is difficult to objectify and subjective. Our analysis is particularly weakened by the brevity of this follow-up period. In addition, an inherent bias is related to the few prospective studies in the literature. Still, it may provide a basis for future research and trials focused on comparing LPS USLS with other surgical techniques for correcting genital prolapse in well-defined sets of patients.

## 5. Conclusion

LPS USLS has been shown to be a technique with a low complication rate and low recurrence rate. In particular, complications related to ureteral damage, which is considered the most frequent complication during vaginal USLS, were minimal. The meta-analysis conducted in comparing these two techniques showed no clear inferiority of one technique over the other. Further, focused and prospective studies will be necessary to confirm this finding.

## Data Availability

The original contributions presented in the study are included in the article, and further inquiries can be directed to the corresponding author.
